# Number Concentration Measurements of Polystyrene Submicrometer Particles

**DOI:** 10.3390/nano12183118

**Published:** 2022-09-08

**Authors:** Paul C. DeRose, Kurt D. Benkstein, Elzafir B. Elsheikh, Adolfas K. Gaigalas, Sean E. Lehman, Dean C. Ripple, Linhua Tian, Wyatt N. Vreeland, Eric J. Welch, Adam W. York, Yu-Zhong Zhang, Lili Wang

**Affiliations:** 1 Biosystems and Biomaterials Division, National Institute of Standards and Technology (NIST), Gaithersburg, MD 20899, USA; 2 Biomolecular Measurement Division, National Institute of Standards and Technology (NIST), Gaithersburg, MD 20899, USA; 3 Protein and Cell Analysis, Thermo Fisher Scientific, Eugene, OR 97402, USA

**Keywords:** bead, calibration, concentration, diameter, flow cytometry, nanosphere, NIST, number, reference materials, submicrometer, standards

## Abstract

The number of techniques to measure number concentrations and size distributions of submicrometer particles has recently increased. Submicrometer particle standards are needed to improve the accuracy and reproducibility of these techniques. The number concentrations of fluorescently labeled polystyrene submicrometer sphere suspensions with nominal 100 nm, 200 nm and 500 nm diameters were measured using seven different techniques. Diameter values were also measured where possible. The diameter values were found to agree within 20%, but the number concentration values differed by as much as a factor of two. Accuracy and reproducibility related with the different techniques are discussed with the goal of using number concentration standards for instrument calibration. Three of the techniques were used to determine SI-traceable number concentration values, and the three independent values were averaged to give consensus values. This consensus approach is proposed as a protocol for certifying SI-traceable number concentration standards.

## 1. Introduction

The detection and characterization of nanoparticles ranging from tens to hundreds of nanometers in diameter have recently inspired increased interest in a wide variety of areas in science, technology, engineering and medicine (STEM). These include (1) virus and bioparticle counting for clinical applications [1,2,3], (2) on-line water bioburden analysis (OWBA) in biopharmaceuticals [4,5], (3) aggregate and insoluble particulate quantitation for protein drug and biotherapeutic efficacy [6,7], (4) particle number concentrations for ultrapure water and chemical solutions in semiconductor manufacturing [8], (5) toxicological and ecotoxicological risks of nanoparticles from commercial products [9], and other areas. These efforts have been driven in part by the recent availability of techniques with improved submicrometer particle detection sensitivity and number concentration determination of liquid suspensions. Several of these techniques are used here.

Micrometer-sized particles have been detected and characterized routinely for several decades using many techniques, such as flow cytometry, optical microscopy and resistive pulse sensing. Over the past 10 years, number concentration measurements with traceability to the International System of Units (SI) have been established for micrometer-sized particles with diameters ranging from 2 μm to 10 μm [10,11,12,13,14]. These techniques include both optical- and electrical-based measurements. In contrast, SI-traceable techniques for determining the number concentration of sub-micrometer particles have only been demonstrated recently and are more difficult to execute than their micrometer-sized particle counterparts, with one technique covering diameters of 600 nm or greater [15] and another limited to 30 nm particles [16]. Here, we target the particle size range from 100 nm to 500 nm in diameter, which is of particular interest in the study of extra-cellular vesicles (EVs) [3], viruses, protein aggregates [6,17] and other bioparticles. Fluorescent particles were chosen here to focus on the development of reference spheres for the calibration of techniques using fluorescence detection, such as flow cytometry. Currently, two commercially available submicrometer calibration materials are on the market for flow cytometry applications [18,19].

Polystyrene microsphere suspensions (with diameter ≥ 2 µm) with known number concentrations are routinely used to characterize instrument performance and act as counting controls for calibration [13,20]. Similar nanosphere suspensions need to be characterized using quantitative number concentration determinations. No techniques have presently been established to determine number concentrations of submicrometer particles (100 nm to 500 nm diameters) with high accuracy and with rigorous traceability to the SI. Traceability to the SI requires that there be an unbroken chain of measurements from primary standards to the measurement of interest, and that the uncertainty of each measurement is quantified and documented. Multiple commercial instruments report number concentration, but these instruments generally rely on user or factory calibration using a sample of assigned number concentration. The underlying problem is that there are no standards available with well-documented uncertainties in this size range, and thus, any instrument relying on an external number concentration standard has poor SI traceability. Without knowing the number concentration uncertainty of the external standard, the overall uncertainty of these methods is difficult to assess.

In this study, exploratory measurements were first conducted with all techniques on common samples of 100 nm, 200 nm and 500 nm fluorescent submicrometer spheres to establish technique-specific protocols. Then, a blinded study was conducted in which the analysts performing the measurements using the established protocols were not informed of the nominal number concentrations beforehand. The results of the blinded study are reported here.

## 2. Materials and Methods

The three types of submicrometer particles measured here are components from a ViroCheck Nanoparticle Reference Kit (Thermo Fisher Scientific, Eugene, OR, USA) with nominal 100 nm (VCN-100), 200 nm (VCN-200) and 500 nm (VCN-500) diameters, referred to as ViroCheck Nanoparticles (VCNs). The ViroCheck spheres were chosen for characterization over other monodispersed submicrometer spheres on the market due to their potential as a complete kit covering all fluorescence channels presently used by commercial flow cytometers (spectral emission wavelengths from 390 nm to 810 nm) and a broad particle size range (diameters from 100 nm to 500 nm). The seven techniques used to measure number concentration and the five techniques used to measure peak diameter are described briefly in the following sub-sections, along with the corresponding methods of determination. More detail is given in the Supplementary Material and references.

### 2.1. Transmission Electron Microscopy (TEM) and Dynamic Light Scattering (DLS)—Particle Sizing

The diameters and size distributions of the VCNs were measured and calculated before and after fluorescent dye staining using a JEOL (Peabody, MA, USA) JEM-200CX TEM. For VCN-100, a TEM magnification of 7200× was used, and for the VCN-200 and VCN-500 particles, a magnification of 6000× was used. All images were digitally captured using EM-Menu software (Version 4.09.53, TVIPS GmbH, Gauting, Germany). Enough images were collected for each sample so that a minimum of 500 particles could be counted and sized. Grating replica crossed line grids (Electron Microscopy Sciences; Hatfield, PA, USA), certified for size by the manufacturer, were imaged along with SI-traceable submicrometer-sized particles, certified by NIST, to validate the size values given by the manufacturer. Images of grating replica grids were used to calibrate the magnification and determine a micrometer-sized scale bar at each magnification setting. Using these grating replica grids allows for precise determination of all particle diameters in each image taken, regardless of magnification. Images of the grating replica grids and ImageJ software (version 1.50i, NIH, Bethesda, MD, USA) were used to determine the number of pixels per micrometer of the camera for each magnification setting. Each sample image was then processed using the ImageJ software, and the resulting diameters of each particle were analyzed using a spreadsheet.

Hydrodynamic diameters, particle distributions (PdI) and count rates were determined by dynamic light scattering (DLS) using a Malvern-Zetasizer Nano (Malvern Panalytical, Malvern, UK) with a laser operating at λ = 632.8 nm, an avalanche photodiode detector with high quantum efficiency and an ALV/LSE-5003 multiple τ digital correlator electronics system. All nanoparticle samples were diluted to appropriate concentrations for measurement with 0.05 μm filtered deionized (DI) water prior to all measurements. Each sample was run using 3 technical replicates to calculate statistics. The general-purpose data analysis mode was used for each sample on each day.

### 2.2. Dry Mass (DM)—Number Concentration

The mass of an aliquot of each submicrometer particle sample dispersed in 0.2 μm filtered deionized ultrapure (DIU) water was measured using an AG245 analytical balance (Mettler Toledo, Columbus, OH, USA) that was calibrated using SI-traceable reference masses. The sample was dried in an oven at 135 °C, and its mass was then measured again. This procedure was repeated until the mass of the sample remained constant from one heating cycle to the next. This mass was assumed to be the dry mass of the sample. The mean diameter of each particle was determined using TEM, as described above, and the mean volume was calculated using the diameter. Number concentrations in particles per unit volume were calculated for each sample using the dry mass of the fluorescent particle, the mean volume of the fluorescent particle and the literature density of amorphous polystyrene (1.055 g/cm^3^). It is assumed that the density of the particles does not change significantly when fluorophores are incorporated.

Once the number concentrations of all particle samples were calculated, each sample was diluted in multiple steps first to 5.00 × 10^9^ mL^−1^ and second to the blinded stock concentrations of 4.0 × 10^9^ mL^−1^, 1.0 × 10^9^ mL^−1^ and 2.5 × 10^9^ mL^−1^ for VCN-100, VCN-200 and VCN-500 suspensions, respectively, using 0.05 μm filtered 2 mmol/L NaN_3_ in DIU water. All dilutions were carefully measured by mass. For DM, the particle size distribution is traceable to the meter by measuring SI-traceable size standards in the same TEM (see above), and traceable mass concentration measurements through the use of a mass balance calibrated using SI-traceable reference masses.

### 2.3. Flow Cytometry (FCM)

The three VCNs were analyzed with a CytoFlex S flow cytometer (Beckman Coulter Life Sciences, Indianapolis, IN, USA) following the protocol recommended by the VCN manufacturer. Violet side scatter (SSC) from a 405 nm laser served as a trigger for the detection of submicrometer particles and discrimination of the noise. The VCN-100 sphere, labeled with a FITC-like dye, and VCN-200 and VCN-500 multicolor labeled spheres displayed fluorescence signals following 488 nm laser excitation with four spectrally separated emission intensities collected from 505 to 545 nm, 564 to 606 nm, 665 to 715 nm and 750 to 810 nm.

#### 2.3.1. Submicrometer Particle Concentration Measurement Using TruCount (TC) Microspheres as an Internal Counting Standard

TruCount (TC) tubes (BD Biosciences, San Jose, CA, USA) were used as an internal standard for determining absolute particle counts. The number of TC microspheres per tube was calibrated by the manufacturer using the DM method to assure SI traceability and has demonstrated agreement with other primary methods in the micrometer range [11,21]. NIST has also used light obscuration (PAMAS, SVSS Particle Counter) as an SI-traceable technique for micrometer-sized particles [12] to confirm the manufacturer’s value. NIST has observed a bias between TC vials of less than 6%, which is the dominant known expanded uncertainty for the TC internal standard method. The large dynamic range of FCM enables direct calibration of submicrometer sphere number concentration through the measurement of micrometer-sized TC spheres. Note that for determining the number concentration of monodisperse spheres, linearity in signals between the submicrometer and micrometer sizes is not necessary. The buffer used for the measurement was filtered with a 20 nm filter from GE healthcare life sciences. The sample line was carefully cleaned between each sample measurement.

For each blinded VCN sample, PBS buffer and Tween 20 were added to a TC tube by mass, such that the sample for measurement contained 0.1% Tween 20, had a total volume of 0.5 mL and number concentrations of about 1 × 10^5^ mL^−1^ for TC microspheres and of between 0.5 × 10^6^ mL^−1^ and 5 × 10^6^ mL^−1^ for VCNs. The number concentration of the TC spheres is known. The ratio of the number of VCN counts to the number of TC counts and the number concentration of the TC microspheres were used to calculate the number concentration of the VCN samples.

#### 2.3.2. Particle Concentrations Measured Volumetrically

Volumetric counting is an absolute number counting method using a volumetric flow cytometer equipped with a peristaltic pump-based fluidic system. A flow rate of 30 µL/min was set in the software and used for measuring samples. Calibrating the flow rate using the manufacturer’s protocol prior to acquisition was vital to ensure the accuracy of the results, where the flow rate and signal collection time were used to calculate sample volume. Flow rate is calibrated by collecting the sample that flows through the flow cytometer during a known time period and measuring its mass using an analytical balance (Mettler Toledo: Columbus, OH, USA) that was calibrated with SI-traceable reference masses. 

ViroCheck spheres were diluted as mentioned earlier, and the measured positive events per unit volume were used for calculation. The largest uncertainties in the volumetric flow cytometry technique are systematic biases that can cause some particles to not be counted. These biases include adsorption of particles on surfaces, non-uniformity in the sample distribution along the fluidic sampling system, particle aggregation and coincidence error. Based on our experience with other flow techniques such as light obscuration and discussions with the manufacturer, we estimated that these types of bias add an expanded uncertainty of 5% or less. Experimental determinations of these biases are planned for future study. The uncertainty in sample volume is estimated to be relatively small in comparison.

#### 2.3.3. Submicrometer Sphere Size Distribution—Mie Scattering Calculation

Estimation of the submicrometer sphere diameters was performed using the side scattering signal from a 405 nm laser in a Mie scattering calculation [22,23]. Flow cytometric results from VCN suspensions with known concentrations and sizes were used for establishing dependence on diameter, wavelength and index of refraction. Then, the Mie scattering procedure was applied to calculate the size distribution of VCN suspensions with unknown concentrations and sizes.

### 2.4. Fluorescence Microscopy (FM)—Number Concentration

The basis of the fluorescence microscopy method is to collect submicrometer spheres from a well-defined, coin-shaped volume of sample onto a charged surface, and then to count the spheres with a standard fluorescent microscope. Immobilization of the spheres on the charged slide forming the lower surface of the sample volume eliminates Brownian motion and localizes the spheres in an easy-to-find focal plane. Stock suspensions were diluted gravimetrically with DIU water by a factor of 1000. This method is practical for number concentrations in the approximate range 3 × 10^6^ mL^−1^ to 3 × 10^7^ mL^−1^. For FM, the dimensions of the interrogated sample volume are traceable to the SI through measurements of calibrated stage micrometers (lateral dimensions) and calibrated gage blocks (vertical dimension).

Slides less than 6 months old precoated with polylysine were rinsed with DIU water and dried (Globe Scientific, Inc., Mahwah, NJ, USA). Adhesive slide wells (Electron Microscopy Services, Hatfield, PA, USA) formed coin-shaped volumes with the coated slides as the bottom face. Distribution of spheres across the surface of the slide was optimized by adding 0.01% by mass of Triton X-100 to the diluted samples. Samples for measurement were at concentrations ranging from (1 to 5) × 10^6^ mL^−1^. After pipetting in 20 µL of sample to slightly overfill the volume, #2 cover slips were placed on the slide wells and sealed with nail polish. Spheres travel slowly to the bottom slide face by both Brownian motion and sedimentation. A well height of 200 µm is large enough to give relatively low uncertainty in the volume height determination, but small enough that spheres will find the bottom face after waiting overnight, as observed and consistent with calculations [24]. The fluorescent signals of the spheres were determined the next day with a standard fluorescence microscope using epifluorescent illumination through a 40×/0.65 NA objective with an arc lamp source and collection through appropriate filters. A cooled scientific cMOS camera acquired 12-bit digital images (Zyla 5.5, Andor Instruments, South Windsor, CT, USA). A calibrated stage micrometer was used to determine the pixel size. Actual measurements of the spheres were made by two scans, in x and y directions. The FIJI variant of ImageJ was used to background subtract, normalize [25] and analyze the images and obtain particle counts [26].

The well area was sampled by performing two scans, one in the x direction and one in the y direction, across the full 1 cm diameter of the well. We fit a parabolic function to the number of particles per image to account for any possible non-uniformity of the sphere deposition across the surface and then used that function to predict the average number concentration across the full well area. The correction factor for deviation from a uniform deposition was moderate (within 7% of unity) for individual samples with the recommended level of surfactant added. Furthermore, the correction factor for measurements in eight separate wells averaged 0.99 ± 0.02 (mean ± SD), demonstrating good average deposition uniformity. The scans in the x- and y-axis directions are correlated because of possible correlations of well height and shifts in concentration during filling. Type A uncertainty of the reported mean number concentration was obtained by taking the standard deviation of number concentrations from the set of two x and two y scans, and dividing by the square root of the number of slide assemblies (two). Uncertainties quoted include a 1.3% Type B relative standard uncertainty for systematic effects in determining the height of the fluid volume.

### 2.5. Particle Tracking Analysis (PTA)

Particle tracking analysis (PTA) is a technique that is used to measure the number and size of particles in a liquid suspension based on light scattered by individual particles. The sample is illuminated with one or more lasers, and videos are recorded to track the changes in particle positions over time. While particle count is based on the number of discrete particles scattering light within a given volume of the measurement, sizing relies on tracking the Brownian motion of a particle over time. To track the Brownian motion, the change in each particle’s position is measured frame by frame. The distance traveled between frames for a particle is used to calculate the mean squared displacement, which can be used to calculate the diffusion coefficient. By applying the Stokes–Einstein (which assumes a spherical particle) equation to the calculated diffusion coefficient, the sphere-equivalent hydrodynamic diameter of the particle is calculated. More detailed information about PTA can be found in the literature [27].

PTA measurements were collected using a ZetaView PMX-110 (Particle Metrix GmbH, Ammersee, Germany) instrument. This instrument used a laser with λ = 405 nm to illuminate the sample. Videos were recorded with a CMOS camera (640 px × 480 px) through a 10×/0.30NA objective. Videos were analyzed using ZetaView software (version 8.04.02 SP2, Particle Metrix GmbH, Ammersee, Germany). The volume of the measurement, used to calculate particle number concentration, is set by the analysis software, so the uncertainty in volume is dependent on the manufacturer’s calibration. The as received stock suspensions of the VCNs, which had number concentrations ranging from (1 to 5) × 10^9^ mL^−1^, were diluted gravimetrically with DIU water (resistivity 18.2 MΩ·cm^−1^, filtered through a 0.2 µm microfiber filter) before measurements were taken. Dilution factors ranged from approximately 20 to 155 times. Submicrometer sphere diameters are reported as the mean of the measured median particle hydrodynamic diameters. Expanded uncertainties for both diameter and particle number concentration are 2× (standard deviation) for n = 15 (100 nm, 500 nm spheres) or n = 17 (200 nm spheres). Two extra measurements were made for the highest dilution 200 nm sphere sample to compensate for the relatively low number of particles measured. To facilitate comparison with the other methods, a single value for sphere size is used (the average of the median diameters reported for each measurement), though we note that the PTA generates a particle size distribution.

### 2.6. Microfluidic Resistive Pulse Sensing (MRPS)

Resistive Pulse Sensing (RPS) is an electrical sensor-based technique used to measure the size and number of particles in a liquid suspension [28]. Particles suspended in a conductive liquid are sensed by electrodes placed on either side of an orifice or constriction. Every time a particle passes through the orifice, it causes a measurable change in the electrical resistance of the constriction, referred to as a “resistive pulse”. The magnitude of the resistive pulse is directly proportional to the volume of the particle. The diameter of the particle is calculated by assuming that all the particles are spherical. Each pulse is counted as a particle event. The number of events is measured over a set sampling time. The volume of sample passing through the orifice during the sampling time must be known to determine the number concentration of the sample. In the case of Microfluidic Resistive Pulse Sensing (MRPS), used here, the sample flows through a microfluidic channel with a nanoconstriction as an orifice, allowing the detection of sample diameters down to 65 nm using 5 μL sample volumes.

MRPS measurements were collected using an nCS1 (Spectradyne, Signal Hill, CA, USA) instrument. The sample holder and microfluidic chip are contained in disposable polydimethylsiloxane cartridges (Spectradyne, Signal Hill, CA, USA). More specifically, the C-400 (65 nm to 400 nm diameter size range) and C-900 (130 nm to 900 nm diameter size range) cartridges were used here. Each lot of cartridges was pre-calibrated by Spectradyne for size and number concentration. A running buffer (200 nm filtered) of PBS (phosphate buffer saline) with 1% (*v*/*v*) polysorbate 20 (Tween^®^20, Sigma-Aldrich Inc., St. Louis, MO, USA) was used in the post-nanoconstriction flow channel to enable appropriate flow and washing. The measured samples were made by diluting the stock suspensions with PBS and the appropriate amount of polysorbate 20, such that the samples contained 1% (*v*/*v*) polysorbate 20 at number concentrations between 5 × 10^8^ mL^−1^ and 3 × 10^9^ mL^−1^. The diluent was put through a 200 nm filter, followed by a 20 nm filter before using. Collected data were analyzed using nCS1 Data Analyzer Software (version 2.5.0.297, Spectradyne LLC, Signal Hill, CA, USA).

The number concentrations measured for VCN-200 and VCN-500 spheres using C-900 cartridges are reported in Table 1 without calibrating with a standard, beyond the one used by the manufacturer to calibrate their C-900 cartridges. The bias in these values relative to those techniques with a stronger SI traceability chain (DM, FCM and FM) are within estimated uncertainties for VCN-200 and VCN-500 suspensions. In contrast, the number concentrations measured for VCN-100 and VCN-200 using C-400 cartridges differed from the higher confidence values by more than a factor of four, being about 400% greater. This convinced us that a standard had to be used to calibrate number concentration values when using the C-400 cartridges. The number concentration of the VCN-200 suspension measured with the C-900 cartridge was used to determine a correction factor for the number concentrations measured with the C-400 cartridges by assuming that the C-900 cartridges gave the correct value. The correction factor was then applied to the number concentrations measured for the VCN-100 suspensions using the C-400 cartridges.

### 2.7. Particle Asymmetric Flow Field Flow Fractionation–Multi-Angle Light Scattering (AF4-MALS)

An Eclipse DualTec AF^4^ separation system (Wyatt Technology Corp., Santa Barbara, CA, USA) was interfaced to an Agilent HPLC system (Model 1260, Agilent Technologies, Santa Clara, CA, USA) including a UV/Vis diode array detector (Model 1260, Agilent Technologies, Santa Clara, CA, USA), a HELEOS-II multiangle light scattering instrument (HELEOS-II, Wyatt Technology Corp., Santa Barbara, CA, USA) and a differential refractive index detector (Optilab T-Rex, Wyatt Technology Corp., Santa Barbara, CA, USA). The separation channel was a vendor-supplied “short” channel with a Mylar^®^ 250 μm thick “wide” spacer, and a 10 kDa nominal-molecular-weight cutoff Ultracel^®^ regenerated cellulose (Millipore, Burlington, MA, USA) ultrafiltration membrane served as the accumulation wall. Samples were introduced into the AF4 separation channel via an autosampler (Model 1260, Agilent Technologies, Santa Clara, CA, USA) with a focus position of 12% of the channel length. The focusing was accomplished by flowing 0.2 mL/min of buffer into the channel inlet and 1.3 mL/min of buffer through the channel outlet for 5 min. After the samples were introduced and focused against the ultrafiltration membrane, they were eluted from the column in a size-selective manner with a channel flow of 1.0 mL/min while the cross flow was linearly ramped from 3.0 mL/min to 0 mL/min over 45 min. Post-separation, the channel was rinsed for 5 min with 1.0 mL/min channel flow and 0 mL/min crossflow and the injector “on” to rinse out the sample loop. The fluid medium for the separation was an isocratic solution of 25 mmol/L sodium chloride (ACS grade, Fisher Scientific, Waltham, MA, USA) and 0.1% (*w*/*v*) sodium dodecyl sulfate (Sigma-Aldrich Inc., St. Louis, MO, USA) with 3.07 mmol/L sodium azide (Ricca Chemical, Arlington, TX, USA).

The eluted fractions flowed into the HELEOS-II detector, where the flow cell was illuminated with a plane-polarized laser (λ = 662 nm) and the scattering intensity was measured at 16 different angles simultaneously. Data analysis and calculations were performed using ASTRA software (version 7.3.2, Wyatt Technology Corp., Santa Barbara, CA, USA). The scattering intensities, scattering angles and laser wavelengths were used to determine the diameter of the monodisperse, spherical particle [29]. The volume of the particle and the refractive indices of the particle and the medium were used to determine the particle number count in the scattering volume. The volume of the flow cell and the time needed for a population of particles to pass through the flow cell were used to calculate the volume passing through the flow cell. This volume and the number of counts in the population were used to calculate the number concentration of a sample.

### 2.8. Reengineered Non-Sorting Analytical Flow Cytometer—Virus Counter (VC)

Unlike traditional flow cytometry, which generally uses a forward scatter trigger based on size as a criterion, the Virus Counter (VC) 3100 instrument (Sartorius Stedim Biotech, Gottingen, Germany) is a reengineered non-sorting analytical flow cytometer that only uses a fluorescence trigger to register an event. The size of viruses is typically 10 nm to 300 nm. The VC was specifically developed to directly quantify virus particles stained with a mixture of a protein and a nucleic acid dye [30]. A yellow laser beam irradiates a narrow sample stream within a transparent flow cell. Optics are used to collect fluorescence from stained particles as they pass through the laser probe region. A dichroic mirror is used to separate the two fluorescence channels. Bandpass filters spectrally filter and isolate each channel’s fluorescence. The fluorescence emissions are detected by photomultiplier tubes (PMTs). Threshold values are automatically determined during sample acquisition. Only the events that occur simultaneously on both channels indicate the presence of co-localized protein and nucleic acid which define virus particles. Data collected from the PMTs are processed in real time to convert the observed fluorescence information into a quantitative measure of virus particle number concentration. Although the instrument was designed for the measurement of virus particles, it can also measure submicrometer spheres with similar fluorescent properties.

The VCN-200 and VCN-500 spheres contain wide wavelength ranges of fluorescence signal excitation/emission profiles, which fit the VC instrument configurations. The VCNs were analyzed with a fluorescence signal from one of the fluorescence channels serving as a trigger for the detection. The samples for measurement were diluted from the stock suspensions in the same way as for conventional FCM with concentrations ranging from 0.5 × 10^6^ mL^−1^ to 5 × 10^6^ mL^−1^. The instrument performance was verified to be ready to measure number concentration within its specifications prior to measuring samples by running a Performance Verification Standard (Sartorius Stedim Biotech) that came with the instrument. This standard is made up of hard-dyed fluorescent spheres in suspension. Unknown concentrations of VCNs were diluted in 20 nm filtered PBS. Samples were tested in triplicate. Data were reported as particles per milliliter.

## 3. Results

Table 1 reports the number concentration values and estimated total expanded uncertainties for the submicrometer particle suspensions measured in particles per milliliter (mL^−1^) using each of the seven techniques (also see Figure S2 for graphic presentation of the results). The expanded uncertainties reported for number concentration include the standard deviation of the measured data and whenever possible, based on our present understanding of the technique, systematic uncertainties. The measured diameters of the submicrometer spheres and standard deviations are also reported in nanometers (nm) in Table 1 for techniques with this measurement capability (also see Figure S3 for graphic presentation of the results).

### 3.1. Number Concentration

DM, FCM and FM give consistent number concentration values for all three submicrometer sphere diameter suspensions and are within about 10% of each other (see Table 1). These three methods also have reliable traceability to the SI. Therefore, the number concentration values from these three techniques were averaged to give a consensus value, with a higher level of confidence in the value and uncertainty than any of those obtained from a single technique. A breakdown of the statistical (type A) and systematic (type B) uncertainties for each of the three consensus techniques is given in Table 2. The number concentration values from the TruCount and volumetric methods are averaged to produce a single FCM value before a consensus value is determined.

**Figure 1:**
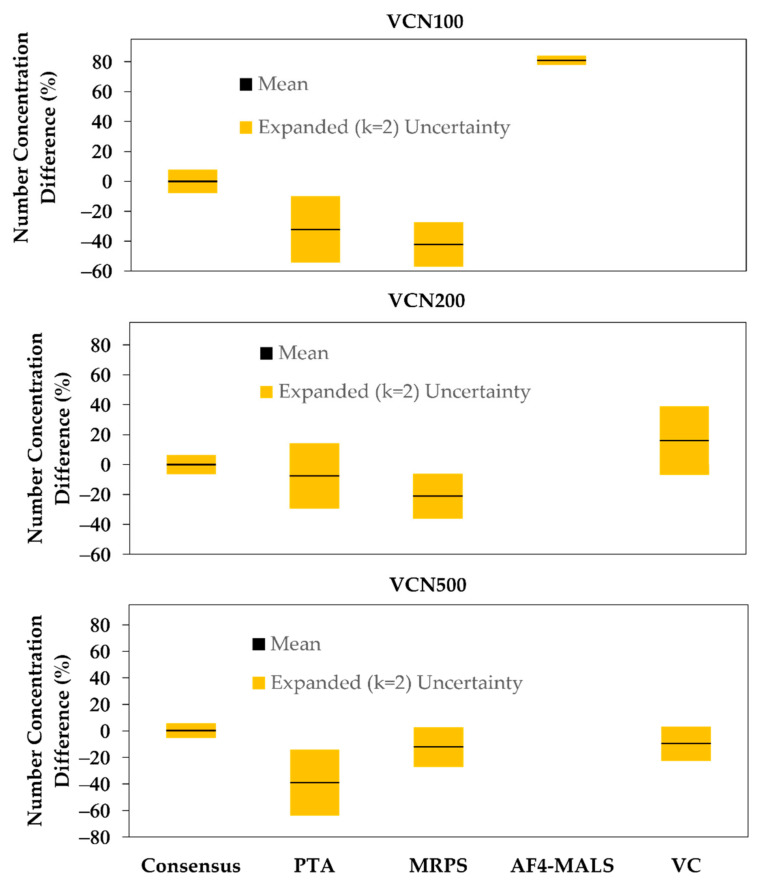
The mean percent difference in measured submicrometer number concentration for each technique relative to the consensus value for the VCNs. The error bars show the estimated relative expanded (*k* = 2) uncertainties for the consensus and technique values.

The percent difference (% Diff) from the consensus value for number concentration using each of the other four techniques is plotted in Figure 1. The corresponding relative expanded (*k* = 2) uncertainty for each technique relative to the consensus value is shown as an error bar for each point. The combined relative expanded uncertainty for number concentration (not shown in Table 1) was calculated by expressing the estimated total uncertainty from Table 1 in terms of percent uncertainty and then adding in quadrature the uncertainty in the consensus value to it. If the combined relative expanded uncertainty is greater than or equal to the % Diff, then the measured values are accurate within the uncertainties. If the combined relative expanded uncertainty is less than the % Diff, and if the uncertainty bars in Figure 1 for the consensus and technique values do not overlap on the y-axis, then an unknown systematic bias is likely to exist for the technique (also see Table S5).

For VC, the number concentration values determined for the 200 nm and 500 nm suspensions are within the uncertainties. VC was not able to measure number concentration for the 100 nm spheres. The number concentration value determined using PTA is within 10% of the consensus value for the 200 nm spheres, but not for the 100 nm and 500 nm spheres where a % Diff of more than 30% was observed. For MRPS, the number concentration value determined for the 500 nm suspension is within the uncertainties, but the values for the 100 nm and 200 nm suspension are not. For AF4-MALS, the number concentration measured for the 100 nm spheres displays a bias from the consensus value that is greater than the estimated uncertainties.

### 3.2. Diameter

**Figure 2:**
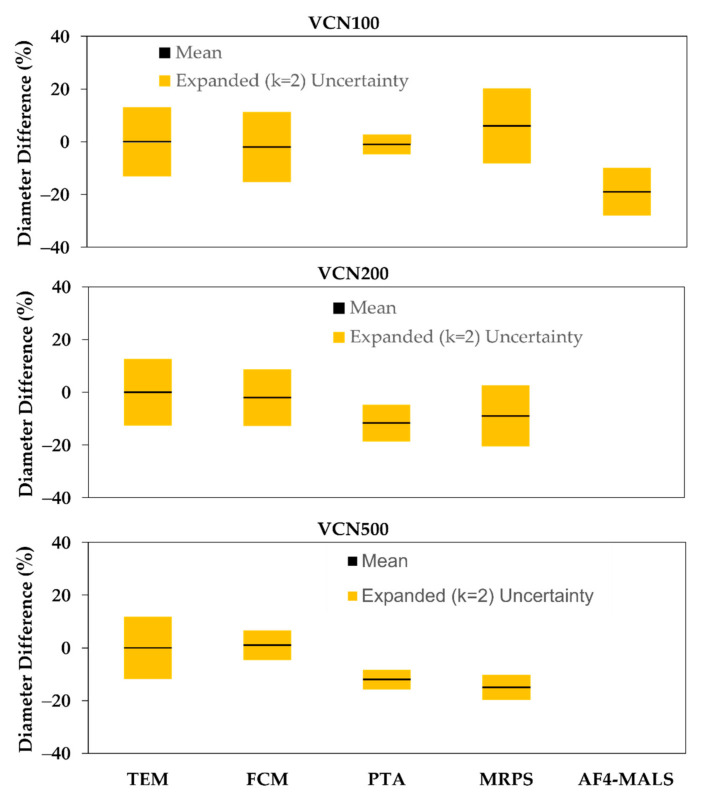
The mean percent difference in measured bead diameter for each technique relative to the TEM value for the VCNs. The error bars show the estimated relative expanded (*k* = 2) uncertainties for the TEM and technique values.

The TEM values measured for mean submicrometer sphere diameters were used as the highest confidence values, and the percent differences of values measured using the other four techniques are plotted in Figure 2. The corresponding percent uncertainty for each technique relative to the TEM value is shown as an error bar for each point (also see Table S5). Mean diameters are compared here and not size distributions, because the distributions were found to be qualitatively symmetrical. In addition, the diameter values calculated from the mean, median and peak are all within ≈1% for each of the samples and techniques, which indicates limited impact from any asymmetry in the size distribution. Differences in diameters between fluorescently labeled and non-fluorescent spheres were found to be insignificant using TEM. This implies that density differences are also insignificant.

The combined relative expanded uncertainty for size was calculated by expressing the standard deviation from Table 1 in terms of percent uncertainty and then adding in quadrature the percent uncertainties for the consensus value (TEM) and the technique of interest. If the combined relative expanded uncertainty is greater than or equal to the % Diff, then the measured values are accurate within the uncertainties. If the combined relative expanded uncertainty is less than the % Diff, and if the uncertainty bars in Figure 2 for the TEM and technique values do not overlap on the y-axis, then an unknown systematic bias is likely to exist for the technique. For FCM, the % Diff of the calculated submicrometer sphere diameters from the TEM values, ranging from 3% to 7%, are well within the estimated uncertainties.

The observed bias in diameter using PTA was about 1% and 7% for the 100 nm and 200 nm spheres, respectively, but increased to a value larger than the uncertainties for the 500 nm spheres. For MRPS, the diameters determined for the 100 nm and 200 nm spheres are also within the uncertainties, but the value for the 500 nm sphere is not. For AF4-MALS, the diameter measured for the 100 nm spheres displays a bias from the consensus value that is greater than the estimated uncertainties. AF4-MALS was not able to measure size or number concentration for the 200 nm and 500 nm diameter spheres due to sample problems related with the presence of fluorophores in the spheres, making the particles ill-behaved during the various fluid manipulations the separation technique requires. Note that FM and VC are optical techniques that do not measure size, so N/A (not applicable) is used to denote this in Table 1.

Particle distributions and diameters were measured by DLS, supporting the TEM data. DLS was also used to track the derived count rate in kilo counts per second (kcps) in order to assess colloidal stability over time. If the particle diameter does not fluctuate over time, then the count rate as measured by DLS can be utilized for monitoring particle concentration. Both the particle diameter and derived count rates remained steady over a 15-month to 2-year period, indicating that these nanoparticle samples are indeed colloidally stable when properly stored (2–8 °C in the dark). In addition, the mean number diameters, which compensate for the DLS increase in intensity with size, determined by TEM (dry-state) and DLS (hydrated-state), were comparable. This is expected as these fluorescently labeled polystyrene particles are stabilized through electrostatic charges near the particle surface rather than using a thick hydrated surface coating to colloidally stabilize the particles, which would have been apparent in DLS and not TEM. Measured mean intensity diameters are indeed larger than those determined by TEM as intensity values are heavily weighted (radius)^6^ towards particles of larger diameters within the distribution.

## 4. Discussion

### 4.1. Number Concentration

The number concentration values determined using DM, FCM and FM techniques were averaged to give a consensus value because traceability to the SI can be demonstrated for each, the uncertainties related with each are relatively well understood, the measured values differed between the three by less than their combined uncertainties and none of these techniques have been shown to give the most accurate values with the smallest uncertainties relative to the other two techniques. None of these criteria for determining confidence can be justified for the other four techniques not included in the consensus determination. 

Electron microscopy can give accurate size measurements of nanoparticles. A mass- or volume-based mean diameter determined for the particle population can be combined with gravimetric determinations of the mass concentration of particles in the liquid suspension, resulting in a traceable number concentration. Two difficulties with this method are that (a) the volume-based mean diameter is sensitive to the fraction of material found as oversized particles, and (b) the relative uncertainty in number concentration is proportional to three times the relative uncertainty of the mean diameter, resulting in relatively high uncertainties. Nonetheless, we have included this method because it is commonly used for commercially available polystyrene particles and because it has a long history of use for number concentration determination.

Alternatives to electron microscopy can directly count particles in a known volume of fluid. Optimized flow cytometers capable of detecting fluorescent particles down to 100 nm diameter are now available that can be either calibrated with larger-size standards of known number concentration or that can determine the measured sample volume directly. This method shows great promise for routine measurements of fluorescent nanoparticle number concentration. We also report on another optical method for direct counting of particles by fluorescence microscopy. In this method, electrostatically charged microscope slides capture particles from a known fluid volume, and the particles are directly measured in a fluorescence microscope.

The values from these three methods with stronger traceability chains are averaged to give a consensus value. We propose that a consensus approach be used to certify number concentration values for submicrometer particles, because even these three techniques have not been characterized and quantified to the extent needed to choose one as a completely reliable SI-traceable technique for particles in this size range. Systematic errors that are not yet well understood for each technique can create significant bias and inaccuracy. Using a consensus approach ensures that all measurement bias and variance have been considered, enabling the determination of a reasonable estimate of the expanded uncertainty at a 95% confidence level.

The consensus-based protocol proposed here for the determination of certified values and uncertainties for number concentration of submicrometer particles is based on NIST protocols using consensus to achieve a higher level of confidence in the accuracy of experimentally determined values and uncertainties than those from any single technique. We consider this study to be just the first step in establishing number concentration standards in this size range. We intend to improve the protocol and further characterize the VCNs before certifying them as standards. In this work, we used an adaptive weighted average model [31] to determine the consensus uncertainties. There are other statistical models for determining consensus uncertainties that are recommended for use depending on the particular characteristics of the data sets [32]. Consultation with our statisticians is needed to determine which model is best for each sphere type’s data going forward as we collect larger data sets. We also intend to study systematic uncertainties for the three consensus techniques in more detail. The stability of the number concentration and diameter of the VCNs demonstrated with TEM and DLS data over more than 15 months is an important characteristic for any potential standard. These measurements will continue to be collected and some of the other techniques used in this study, fluorescence-based, in particular, will be added going forward to continue monitoring stability.

We now discuss the possible causes of bias that can explain why the values for the non-consensus techniques differ from the consensus values by more than the estimated combined uncertainties. The particle number concentration values obtained for PTA are highly dependent on image acquisition settings (e.g., shutter speed and camera gain), analysis settings (e.g., brightness threshold, scattering area limits), and particle scattering characteristics. Uncertainties related to these parameters are not well defined and are not included in the reported uncertainties for the values in Table 1, which include only the standard deviations across five dilutions and multiple days. It is also important to note that sample volume is determined by manufacturer calibration, the details of which are proprietary, which allows concentration to be calculated using the measured particle counts. Therefore, the accuracy of their volume calibration will directly affect the accuracy of the measured number concentration values. The reported number concentration values are also subject to relatively large extrapolation factors of the order of 2 × 10^4^, owing to the small volume of sample illuminated by the laser and captured by the camera (≈50 nL for each measurement). Because a certified standard for particle number concentration for spheres in this size range is not yet available, users can estimate the best settings for a particular sphere population based on suspensions with provider-supplied number concentrations when available, or they can estimate the best settings for a particular sphere population based on image characteristics [33]. In this study, the results indicate that the camera settings were well matched, by chance, to the scattering characteristics for the 200 nm sphere, but were insufficiently sensitive to accurately capture the number concentrations of the other two sphere samples (VCN-100, VCN-500). In particular, a higher camera gain level would need to be used for the VCN-100 and VCN-500 spheres, but the specific gain level needed to obtain accurate number concentration values is difficult, if not impossible, to determine without using number concentration standards to optimize the settings. With well-established number concentrations (for example, in a potential follow-up round using the consensus values for the submicrometer spheres characterized in this study), it would be feasible to establish appropriate settings (e.g., camera gain levels) for samples featuring particles with similar scattering characteristics. A recent study to quantify gold nanoparticles included PTA and demonstrated a possible approach to quantify uncertainties [34].

The number concentrations of the submicrometer sphere suspensions measured with MRPS were consistently smaller than the consensus value with the bias for the 500 nm sphere within the estimated uncertainties, the 100 nm bias well beyond the uncertainties and the 200 nm bias approximately equal to the uncertainties. MRPS is another technique where the instrument manufacturer relies on number concentration standards to calibrate the instrument, or in this case, the sample cartridges, for volume. Thus, again, the accuracy of their calibration standards will directly affect the accuracy of the measured number concentration values. The negative bias we observe in the data may be corrected by using more accurate number concentration standards, which could be used to calibrate a cartridge lot or even individual cartridges. The increasing bias with decreasing diameter shows that number concentration standards with multiple diameters close to those of analytes are needed. Some indication of changes in bias with number concentration was also observed, based on preliminary results, so an optimal concentration range with the smallest concentration dependence also needs to be explored further.

Particle number concentrations calculated from MALS measurements after AF4 separation differ significantly from the corresponding consensus values for VCN-100 and were not even able to be measured for VCN-200 and VCN-500. These difficulties are both likely due to the presence of the fluorescent molecules within the VCNs. The calculation of particle number concentration with MALS measurements requires accurate knowledge of both particle volume (from particle shape and size) and particle refractive index, as shown in Equation (S4) (particle count from MALS data); thus, any inaccuracy in particle size measurement from MALS calculations or variations in the refractive index of the particle from the presence of the fluorophore will obfuscate and complicate these measurements, substantially increasing the inaccuracy of the measurement. Number concentration measurements of non-fluorescent polystyrene submicrometer spheres have been measured routinely using the same instrumentation and have also been reported in the literature [35]. Since the MALS measured particle size is smaller than the TEM value, it follows that the measured particle number concentration is greater than the consensus value, which is what is observed. Fluorescence is a problematic interference that is very difficult to separate from the scattered light signal. For VCN-100, scattering was the main contribution to the signal, with fluorescence only causing background interference, since the fluorescence from the FITC-like dye is green and the orange-red laser (662 nm) did not produce much fluorescence. For VCN-200 and VCN-500, the fluorescence contribution to the signal was comparable to that of scattering, making size and counting measurements impossible. In contrast to number concentration, determining sample volume using AF4-MALS is relatively easy. It is calculated using the flow rate, signal collection time and the volume in the flow cell illuminated by the laser. As a result, the calculated sample volume will not change based on the particle size, composition or the presence of fluorescence.

Using VC, the VCN-100 spheres could not be measured, due to their very dim fluorescence. This is because VC only has one yellow laser and its detection system uses fluorescence as a trigger. The weak signal could not be discriminated from the background, resulting in a failure to detect the spheres.

### 4.2. Diameter

For size measurements, TEM has been shown to be highly accurate with small uncertainties, and the other four techniques have not. Therefore, the diameters determined from TEM were used as the highest confidence values, which were compared to the corresponding values of the other four techniques. Here, we explore the possible causes of bias that can explain why some of the corresponding values differ from the TEM value by more than the estimated combined uncertainties.

Note that FM and VC use optical detection, which does not measure size. In addition, the size calculation used with the FCM data cannot be used with the VC data. This is because the Mie scattering calculation predicts a diameter value using the SSC profile. VC counts an electric pulse when a trigger signal is above the background. VC measurement does not generate FSC/SSC profiles like typical flow cytometers do. Since VC only counts electric pulses without double scattering profiles, VC cannot be used to determine the particle diameter.

Sizing in PTA depends on tracking the motion of particles, with larger particles moving more slowly. For a typical analysis, the particle size can be reported as the mean, mode (peak), or, as reported here, the median. The median and modal values are equally robust, and the PTA method may be validated for size determination of particles, with a study showing relative expanded uncertainties for sizing of nanoparticles (*d* ≤ 100 nm) from ≈(10 to 14)% [36]. For larger particles, their relatively slower motion can be compensated for by slowing the camera frame rate (see Methods), but there are also increased uncertainties in identifying the center of mass for larger particles, which can bias the analysis toward smaller particle sizes [27]. The combination of these factors leads to increasing bias/uncertainty in the calculation of diameter values with increasing particle size, beyond what is captured in the reported measurement-to-measurement standard deviations.

The bias in diameter values measured using MRPS was found to increase with increasing diameter. The 100 nm and 200 nm diameter values agreed with the TEM values within the estimated uncertainties, whereas the 500 nm sphere diameter values did not. This may also be a calibration issue, where standard diameter spheres need to be used with multiple diameters close to those of the analytes. 

Particle diameters calculated from AF4-MALS measurements differ significantly from the corresponding TEM values. Again, this is likely due to the presence of the fluorescent molecules within the spheres. Size measurements of non-fluorescent polystyrene submicrometer spheres have been measured routinely using the same instrumentation and have also been reported in the literature [37]. In the sub-micrometer diameter size range, the scattered light is 0.01 ppm to 100 ppm relative to the incident light source. The very low scattered light intensity (as little as 10^−8^ relative to the incident light intensity) requires careful design of the detector geometry so spurious scatter and reflections from various components of the detector flow cell hardware (windows, walls, etc.) do not contaminate light scattered from the sample reaching the individual discrete MALS detectors. The presence of fluorophores complicates this analysis. Even fluorophores with maximum excitation efficiencies at wavelengths significantly shorter than scattering incident light sources (usually around 660 nm in modern instruments) can still fluorescently emit an amount of light equivalent to the light scattered by these small particles. To avoid these complications, narrow bandpass filters are needed to block the red-shifted fluorescently emitted photons from reaching the MALS detectors. These filters are not in the instrument used in these studies, which results in the observed data. Thus, the MALS-determined particle sizes are not as accurate as possible with the MALS instrument designed to work with fluorescent materials.

## 5. Conclusions

We used a multiple-technique approach to determine consensus values for number concentrations of submicrometer particle suspensions with greater accuracy and smaller uncertainties than achievable with any single technique. Each of the three techniques used for consensus yielded SI-traceable values that are independent of those from the other consensus techniques in both concept and uncertainties, enabling the observed improvement. This approach will aid in the development of number concentration reference materials in the 80 nm to 1 μm diameter size range, which are greatly needed for quantitation of analytes in many high-impact submicrometer particle and submicrometer bioparticle applications and for improving the accuracy of complementary techniques.

## Figures and Tables

**Table 1:** Measured submicrometer sphere number concentrations and diameters for stock suspensions with expanded (*k* = 2) uncertainties.

Technique	100 nm Sphere		200 nm Sphere		500 nm Sphere	
	Size nm	Concentration mL^−1^	Size nm	Concentration mL^−1^	Size nm	Concentration mL^−1^
TEM and DM	107 ± 14	(4.0 ± 1.6) × 10^9^	189 ± 24	(1.0 ± 0.4) × 10^9^	492 ± 58	(2.5 ± 0.9) × 10^9^
FCM	105 ± 14	(4.4 ± 0.3) × 10^9^ TruCount standard	186 ± 20	(0.90 ± 0.06) × 10^9^ TruCount standard	498 ± 28	(2.6 ± 0.2) × 10^9^ TruCount standard
		(3.9 ± 0.3) × 10^9^ Volumetric		(0.88 ± 0.06) × 10^9^ Volumetric		(2.5 ± 0.2) × 10^9^ Volumetric
FM	N/A	(3.8 ± 0.3) × 10^9^	N/A	(0.8 ± 0.2) × 10^9^	N/A	(2.7 ± 0.2) × 10^9^
PTA	106 ± 4	(2.7 ± 0.6) × 10^9^	175 ± 4	(0.8 ± 0.2) × 10^9^	433 ± 16	(1.6 ± 0.4) × 10^9^
MRPS	113 ± 16	(2.3 ± 0.3) × 10^9^	172 ± 20	(0.7 ± 0.1) × 10^9^	419 ± 20	(2.3 ± 0.3) × 10^9^
AF4-MALS	87 ± 8	(7.2 ± 0.2) × 10^9^				
VC			N/A	(1.0 ± 0.2) × 10^9^	N/A	(2.4 ± 0.3) × 10^9^

**Table 2:** Components of the relative standard (*k* = 1) uncertainty in the measured number concentration for each of the consensus techniques, (a) FCM—internal standard, (b) FCM—absolute volume, (c) FM, (d) TEM—diameter measurement, (e) DM/TEM—number concentration.

Component	Type	*d* = 100 nm	*d* = 200 nm	*d* = 500 nm
(a)				
Repeatability	A	1.0%	1.0%	1.0%
TruCount number concentration	B	3.0%	3.0%	3.0%
Gate choice	B	1.0%	1.0%	1.0%
Background counts	B	1.0%	1.0%	1.0%
Combined rel. standard uncertainty		3.5%	3.5%	3.5%
(b)				
Repeatability	A	1.0%	1.0%	1.0%
Volume determination	B	1.0%	1.0%	1.0%
Gate choice	B	1.0%	1.0%	1.0%
Background counts	B	1.0%	1.0%	1.0%
Other counting errors	B	2.5%	2.5%	2.5%
Combined rel. standard uncertainty		3.2%	3.2%	3.2%
(c)				
Repeatability	A	3.3%	12.6%	2.9%
Sample cell height	B	1.3%	1.3%	1.3%
Pixel size	B	0.2%	0.2%	0.2%
Background counts	B	0.7%	0.7%	0.7%
Combined rel. standard uncertainty		3.6%	12.7%	3.3%
(d)				
Diameter repeatability	A	0.3%	0.2%	0.3%
Diameter accuracy	B	6.5%	6.3%	5.9%
Particle segmentation	B	0.2%	0.2%	0.2%
Combined rel. standard uncertainty		6.5%	6.3%	5.9%
(e)				
Propagated diameter uncertainty	B	20.0%	19.0%	18.0%
Mass repeatability	A	0.5%	1.1%	0.4%
Mass accuracy	B	1.4%	0.4%	0.7%
Combined rel. standard uncertainty		20.1%	19.0%	18.0%

## Data Availability

The data presented in this study are available in Table S5.

## Supplementary Materials

The following supporting information can be downloaded at: https://www.mdpi.com/article/10.3390/nano12183118/s1, Figure S1: VCN submicrometer spheres and TC microspheres measured by flow cytometry; Figure S2: Schematic defining the geometry of the Mie scattering; Figure S3: Mean number concentration of VCNs in stock suspensions, measured using each technique; Figure S4: Mean measured diameters for the VCNs using each technique and TEM; Table S1: Technical data for the Mettler Toledo AG245 Scale; Table S2: FCM gain settings for VCN number concentration measurements; Table S3: Bead concentration measurement in PBS with different amounts of Tween 20; Table S4: Particles counted and tracked by PTA; Table S5: Comparison of percent difference from consensus values to combined expanded uncertainty for measured number concentrations and diameters of VCN suspensions.

## NIST Disclaimer

Certain commercial equipment, instruments and materials are identified in this paper to specify an experimental procedure as completely as possible. In no case does the identification of particular equipment or materials manufacturer imply a recommendation or endorsement by the National Institute of Standards and Technology, nor does it imply that the materials, instruments or equipment are necessarily the best available for the purpose. This manuscript is a contribution of NIST, and therefore is not subject to copyright in the United States.
