# Supplementary material for: Number Concentration Measurements of Polystyrene Submicrometer Particles
Source: Nanomaterials (Basel). 2022 Sep 8;12(18):3118. doi: 10.3390/nano12183118 (PMC9501160; doi:10.3390/nano12183118)
Supplement: Supplementary file 1 [file nanomaterials-12-03118-s001.zip › nanomaterials-1836201-supplementary.pdf]

nanomaterials

MDPI

Type of the Paper: Article

# Supplemental Material:

# Number Concentration and Size Measurements of Polystyrene Submicrometer Particle Suspensions using Several Different Techniques

**Paul C. DeRose [1,*], Kurt D. Benkstein [2], Elzafir B. Elsheikh [1], Adolfas K. Gaigalas [1], Sean E. Lehman [2], Dean C. Ripple [2], Linhua Tian [1], Wyatt N. Vreeland [2], Eric J. Welch [3], Adam W. York [3], Yu-Zhong Zhang [3] and Lili Wang [1]**

[1] Biosystems and Biomaterials Division, National Institute of Standards and Technology (NIST), Gaithersburg, MD 20899, USA

[2] Biomolecular Measurement Division, National Institute of Standards and Technology (NIST), Gaithersburg, MD 20899, USA

[3] Protein and Cell Analysis, Thermo Fisher Scientific, Eugene, OR 97402, USA

* Correspondence: paul.derose@nist.gov

## 2. Materials and Methods

### 2.1 *Transmission Electron Microscopy (TEM) – Submicrometer Particle Sizing*

Each sample was diluted to approximately 0.03 % (w/w) solids using 0.2 µm filtered deionized water. Square 200 mesh copper grids (Electron Microscopy Sciences; Hatfield, PA) were coated with 2 % (w/w) sterile collodion (Paralodion) in amyl acetate. The coated grids were air dried for 5 minutes. Once dry, then 5 µL of each sample was carefully pipetted onto the coated grid and allowed to air dry at room temperature for at least 1h prior to imaging.

### 2.2 *Dry Mass (DM) – Number Concentration*

The ViroCheck Nanoparticles (VCNs) were prepared by loading the polystyrene particles with hydrophobic dyes using a solvent solution that swelled the particles and allowed the dyes to diffuse into the hydrophobic polystyrene matrix. The solvent was then evaporated off, trapping the dyes inside the hydrophobic environment. After

staining, the particles were washed thoroughly with methanol to remove any surface or loosely bound dye.

Thermogravimetric analyses were performed on all VCN samples. Prior to dry mass determination the 100 nm particle (VCN-100) samples were set to approximately 2 w/v% solids and the 200 nm particle (VCN-200) and 500nm particle (VCN-500) samples were set to 1.5 w/v% solids. Dry mass for each sample was determined by first weighing an aluminum weigh pan and then pipetting 200 µL to 350 µL of each sample into the pan. Each weigh pan was placed in a 100°C oven for 2h, removed and weighed and then placed back in the oven for 30 min and weighed again to ensure all the water had evaporated, indicated by no change in the measured weight. Once each sample was completely dry, wt% solids were calculated using w/w% and w/v%. Percent solids by w/w% and w/v% were the same to at least the hundredths place. Dry mass for each submicrometer particle sample was repeated in triplicate. All masses were measured using an AG245 analytical balance (Mettler Toledo: Columbus, OH) that is serviced and calibrated semiannually. Its technical specifications are shown in Table S1.

**Table S1:** Technical Data for the Mettler Toledo AG245 Scale.

| Technical data | AG204 DR® | AG245** | AG285 |
|---|---|---|---|
| Readability | 1 mg/0.1 mg[1)] | 0.1 mg/0.01 mg[1)] | 0.1 mg/0.01 mg/0.01 mg[1)] |
| Maximum capacity | 210 g/81 g[1)] | 210 g/41 g[1)] | 210 g/81 g/41 g[1)] |
| Taring range | 0...210 g | 0...210 g | 0...210 g |
| Repeatability (s) | 0.5 mg/0.1 mg[1)] | 0.1 mg/0.02 mg[1)] | 0.1 mg/0.05 mg/0.02 mg[1)] |
| Linearity [2)] | ±1 mg/±0.2 mg[1)] | ±0.2 mg/±0.03 mg[1)] | ±0.2 mg/0.1 mg/±0.03 mg[1)] |
| Stabilization time (typical) | 3 s | 3 s/15 s[1)] | 3 s/15 s[1)] |
| Adjustment | internal, fully automatic motorized initiation (FACT) and test possibility for checking the sensitivity | | |
| • with internal weight | 200 g | 200 g | 200 g |
| • with external weights | 50/100/200 g | 40/100/200 g | 40/100/200 g |
| Sensitivity | | | |
| • Temperature drift [2)] | ±1.5 ppm/°C | ±1.5 ppm/°C | ±1.5 ppm/°C |
| • Long-term drift [3)] | ±0.003 % | ±0.003 % | ±0.003 % |
| Display | backlit LCD | LCD, not backlit | LCD, not backlit |
| Interface | LocalCAN universal interface | | |
| Weighing pan | ø 85 mm, stainless steel | | |
| Effective height above pan | 240 mm | | |
| Dimensions (w/d/h) balance | 205 x 330 x 310 mm | | |
| Net weight/with packaging | 4.9 kg/7.25 kg | | |

1) Values in the fine range (AG135, AG245, AG285) or DeltaRange (AG204 DeltaRange®)
2) In the temperature range 10 … 30°C
3) Sensitivity deviation/year after first-time startup with self-calibration FACT switched on
** Production phaseout form June 2000

Once the dry mass was determined for each sample, concentrations in particle per milliliter were calculated for all stained particle samples using the following equation:

$$\frac{Particles}{mL} = \frac{\frac{w}{v}\%\ Solids}{100 \times V_{particle}\,\rho_{PS}} \quad \textbf{(S1)}$$

where percent solids were calculated in w/v%, $V_{particle}$ (4/3 $\pi r^3$) is the volume of one particle in $cm^3$ and $\rho_{PS}$ is the density of polystyrene (1.055 $g/cm^3$). The diameters/radii of the submicrometer particle samples were determined by TEM as described above and were converted from nm to cm.

### *2.3 Flow Cytometry (FCM)*

Flow cytometry was originally designed to detect eukaryotic biological cells, which are generally 6 µm or larger in diameter. Standard instruments are therefore not suitable for direct acquisition of submicrometer particles using the same instrument setting for cell detection. Smaller particles scatter less light and have weaker fluorescence signals. To better detect smaller particles, new instruments have been developed that have improved light detection and use a 405 nm violet laser instead of 488 nm blue laser, which results in more light scattering and increases the ability to detect submicrometer sized particles and bioparticles with lower refractive indexes.

ViroCheck NanoParticle Reference Kit (Thermo Fisher Scientific) samples with known bead concentrations and sizes were used to establish proper instrument settings (see Table S2) in specified fluorescence channels and measurement protocols prior to analyses of VCNs with blinded unknown concentrations. All buffers were filtered with a 20 nm filter from GE healthcare life sciences. The sample line was carefully cleaned between each measurement.

**Table S2.** FCM Gain Settings for VCN Number Concentration Measurements.

| Channel | VCN-100 | VCN-200 | VCN-500 | TC |
|---|---|---|---|---|
| **FSC** | 73 | 73 | 73 | 73 |
| **SSC** | 61 | 61 | 61 | 61 |
| **Violet SSC** | 100 | 100 | 100 | 100 |
| **FITC** | 2000 | 2000 | 750 | ----- |
| **PE** | ----- | ----- | ----- | 50 |
| **PC5.5** | ----- | 750 | 250 | ----- |
| **PC7** | ---- | ----- | ----- | 750 |
| **Threshold** | FITC 2000 | Violet SSC 20,000 | Violet SSC 100,000 | |

*2.3.1 Submicrometer particle concentration measurement using Trucount (TC) microspheres as an internal counting standard*

To identify an optimum buffer for the measurement, the VCN-100 spheres were diluted in phosphate buffer saline (PBS) with different amounts of Tween 20 in TC tubes (Table S3). A known amount of VCNs was added to TC tubes in the PBS buffer with different amounts of Tween 20 to make a total volume of 0.5 mL. The dilution factor was calculated by weighing both bead suspension and PBS buffer. Samples were then run on the flow cytometer (Figure S1). After setting appropriate gates for both VCNs and TC microspheres as shown in Figure S1, bead concentrations were calculated using Eq. S1. All samples were run in fast speed flow rate for 2 min with abort rate less than 5%. On the basis of the results of the bead concentration shown in Table S3, PBS buffer with 0.1 % Tween 20 was used as the buffer for bead concentration measurement with TC bead as the internal counting standard. Additionally, events of VCNs and TC microspheres could be obtained from multiple fluorescence channels and hence used for assessing the uncertainty associated with bead concentration measurement.

$$[ViroCheck\ Beads] = \frac{ViroCheck\ bead\ events}{TC\ bead\ events} \times \frac{TC\ beads\ per\ tube}{ViroCheck\ bead\ weight} \quad \textbf{(S2)}$$

**Table S3.** Bead Concentration Measurements in PBS with Different Amount of Tween 20.

| Tween 20 % in PBS | Concentration (mL$^{-1}$) |
|---|---|
| 0.1 % | $9.20 \times 10^{8}$ |
| 0.5 % | $9.08 \times 10^{8}$ |
| 1 % | $9.15 \times 10^{8}$ |
| 0 % | $5.84 \times 10^{8}$ |

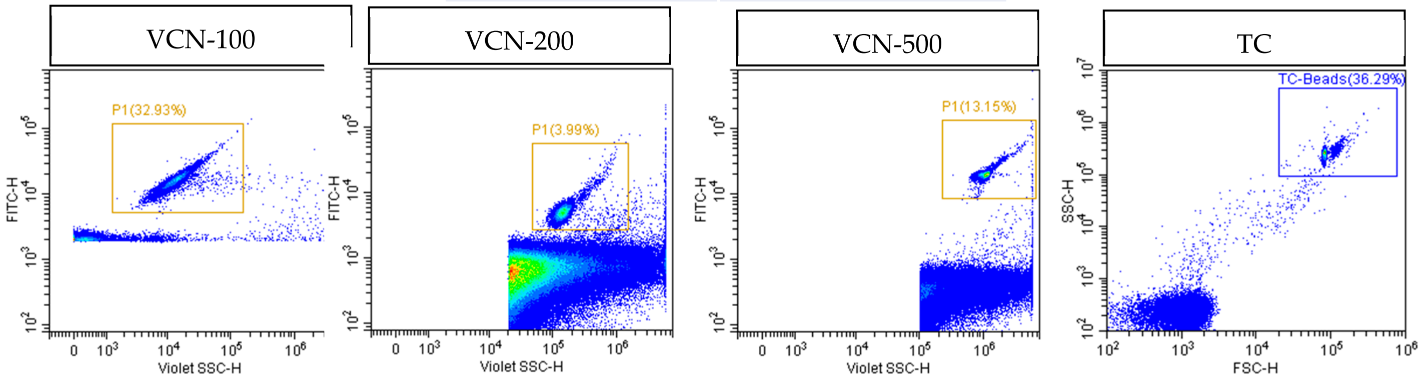


**Figure S1:** VCN Submicrometer Spheres and TC Microspheres Measured by Flow Cytometry.

*2.3.2 Particle concentrations measured volumetrically*

Volumetric counting is an absolute number counting method using a volumetric flow cytometer equipped with a peristaltic pump-based fluidic system. Calibrating the flow rate prior to acquisition is vital to ensure accuracy of the results. VCNs were diluted as mentioned earlier and positive bead events per microliter were used to calculate bead concentration of the stock suspensions, such that VCN concentration = events/μL × 1000 × dilution factor. Gravimetry is used to calibrate the sample volume and assures SI traceability.

*2.3.3 Submicrometer sphere size distribution – Mie scattering calculation*

Estimation of the bead diameters was calculated using a Mie scattering calculation. It used Mie scattering code as summarized in Figure S2 with all angles set to 90°. The functions $S_1$ *a*nd $S_2$ are scattering amplitudes defined and evaluated by the Mie code. Mie scattering theory is rigorously applicable to scattering from homogeneous solid spheres. The calculations were checked using NP with known properties to verify that the calculation reproduces known dependence on diameter, wavelength, and index of refraction. Then the Mie scattering code was applied to calculating scattering from VCNs.

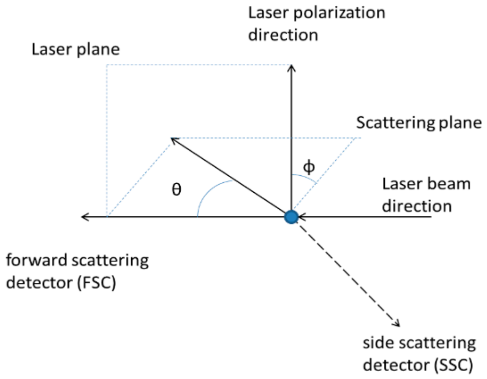


$$E_\theta = \cos(\phi)\, S_2(cos\theta)$$
$$E_\phi = \sin(\phi)\, S_1(cos\theta)$$
$$E^2 = E_\theta{}^2 + E_\phi{}^2$$

**Figure S2:** Schematic Defining the Geometry of the Mie Scattering.

A calibration of the Violet SSC signal versus particle diameter was performed using populations of polystyrene submicrometer particles with known diameters from 100 nm to 500 nm. Next the reference submicrometer spheres were replaced by VCNs and the sizes were calculated.

*2.4 Fluorescence Microscopy (FM) – Number Concentration*

The height of the volume was determined by focusing on faint ink marks, applied prior to assembly, on the underside of the cover slip and the face of the polylysine-coated

slide; the difference in stage travel multiplied by refractive index of the solution is the height. Stage travel was calibrated by measuring pairs of calibrated steel gage blocks (Mitutoyo America, Aurora, IL) wrung to an optical flat. Height measurements of the sample-filled volumes were completed between 10 min and 2 h after sealing. Spheres travel slowly to the bottom slide face by both Brownian motion and sedimentation. A well height of 200 μm is large enough to give relatively low uncertainty in the volume height determination, but small enough that spheres will find the bottom face after waiting overnight.

Actual measurements of the spheres were made by two scans, in $x$ and $y$ directions, on axes slightly displaced from the center of the well so that the ink marks would not interfere with the images. Each scan had between 6 and 12 images. Images were not contiguous to minimize photobleaching. The underside of the coverslip was inspected for adherent spheres; if any were observed, scans of this surface were also included. If spheres were observed to be present in the fluid volume between the two faces, the sample was discarded.

We used the FIJI variant of ImageJ to analyze the images and obtain particle counts. First, the median background intensity was subtracted from all images. Visual inspection showed that the sphere fluorescence was dimmer near the edges of the image. The acquired image was divided into a 6 by 6 grid, and for each section, the measured fluorescence of each sphere was normalized by dividing by the median integrated fluorescence for particles found in that grid section. Simpler methods of normalizing fluorescence images by dividing by a fluorescent image of a uniform white card did not work as well, especially at the image perimeter. Caicedo et al. [1] recommends a similar approach to normalize fluorescence of cell images by retrospective analysis of multiple image frames.

The binary threshold was chosen to minimize number of doublets but to also capture all visible particles. To ensure more accurate fluorescence intensity measurements, particles in the binary image were dilated by 1 pixel. In FIJI, the Analyze Particle feature was used to count particles of the modified binary image while also measuring the integrated fluorescence of the original image using the redirection feature. Fluorescent particles were counted if the fluorescence was greater than 0.4 times the median particle fluorescence.

The Type A uncertainty accounts for statistical fluctuations of the counts and the non-uniformity of particle distribution across the surface. Uncertainties for the gage block heights, stage micrometer, and pixel size determination with the micrometer are all less than 0.2 % and negligible. Further systematic studies of the non-uniformity of particle distribution on the bottom face are desirable to obtain a better evaluation of that uncertainty. We are also developing screening methods for the polylysine-coated slides

and deposition process to ensure that all particles have adhered to the slide faces prior to measurement.

*2.5 Particle Tracking Analysis (PTA)*

Videos were recorded exclusively in scatter mode (no fluorescent particle tracking used). Cell temperatures were set to 25 °C and maintained with the on-board Peltier controller. Instrument performance for sizing was checked against a
110 nm diameter polystyrene bead. Video acquisition and analysis settings were selected based upon expected fluorescent bead diameters (*d* = (100, 200, or 500) nm). In particular, the shutter speed and video frame rate were adjusted for the different bead sizes. Other acquisition parameters, including camera gain, minimum pixel brightness, minimum particle area (in pixels), and minimum track length (in frames), were kept constant across all measurements. Five distinct dilutions were prepared for each stock suspension, and each dilution was measured in triplicate. For each bead sample, the total numbers of particles counted and tracked, along with video acquisition settings, are shown in Table S4. Only the tracked particles provide information on particle size distribution. Diameters are reported as the mean of the median diameters reported for each measurement.

**Table S4:** Particles Counted and Tracked by PTA

| Sample | Shutter | Frame rate (Hz) | Particles counted | Particles tracked |
|---|---|---|---|---|
| 100 nm bead | 100 | 30 | 36834 | 19250 |
| 200 nm bead | 200 | 15 | 16873 | 9092 |
| 500 nm bead | 500 | 7.5 | 19489 | 9567 |

*2.6 Microfluidic Resistive Pulse Sensing (MRPS)*

Size distributions of suspensions of VCN-500 and VCN-200 spheres were collected using C-900 cartridges and number concentrations were determined by integrating over a diameter range from (300 nm to 500 nm) and (142 nm to 300 nm) for 500 nm and 200 nm spheres, respectively. Size distributions of suspensions of VCN-200 and VCN-100 spheres were collected using C-400 cartridges and number concentrations were determined by integrating over a diameter range from (142 nm to 300 nm) and (80 nm to 170 nm) for 200 nm and 100 nm spheres, respectively. The number of events collected (N) and corresponding percent error (%ER) for 500 nm and 200 nm spheres using C-900 cartridges were (N=40,000; %ER= ±0.5%) and (N=20,000; %ER= ±0.7%), respectively. The corresponding values for 200 nm and 100 nm spheres using C-400 cartridges were (N=1,200; %ER= ±3.0%) and (N=1,800; %ER= ± 2.5%), respectively. We wanted to collect

enough events, such that all percent errors were less than ± 1%, but this was not possible for VCN-200 and VCN-100 spheres with the C-400 cartridges due to clogging issues.

*2.7 Particle Asymmetric Flow Field Flow Fractionation-Multi-Angle Light Scattering (AF4-MALS)*

The eluted fractions flowed into the HELEOS-II detector, where the flow cell was illuminated with a plane-polarized laser ($\lambda$ = 662 nm) and the scattering intensity was measured at 16 different angles simultaneously.

$$R(\theta) \propto P(\theta) = \left[\frac{3}{u^3}(\sin u - u\cos u)\right]^2 \quad \textbf{(S3)}$$

$$u = \frac{4\pi}{\lambda} a \sin\frac{\theta}{2} \quad \textbf{(S4)}$$

In equation S2, $R(\theta)$ is the excess Rayleigh ratio measured at angle θ, $a$ is the radius of the spherical particle, and λ is the wavelength of the laser. The slope of R(θ) vs. θ for each measurement volume eluting from the AF4 was used to determine the particle radius $a$, using equations S2 and S3 for each eluting measurement volume. Please note this calculation is only valid where all the particles in an eluting measurement volume are sufficiently uniform in size and composition.

Then the particle number count determined according to Equation S4:

$$R(0) = \frac{8\pi^2}{\lambda^4} N \left(\frac{n_{particle} - n_0}{n_0}\right)^2 V_{particle}^2 \quad \textbf{(S5)}$$

here $R(0)$ is the extrapolated scatter intensity at 0° angle, λ is the laser wavelength, $V_{particle}$ is the volume of the particle, $N$ is the particle number count in each measurement volume, and $n_{particle}$ and $n_0$ are the refractive indices of the particle and the medium respectively. Extrapolation and all calculations were done with ASTRA 7.3.2 software.

AF4-MALS measurements were conducted on the polystyrene latex (PSL) samples obtained from Thermo Fisher Scientific. Samples of 100 nm polystyrene latex with and without FITC (Dyed and No Dye) were measured. Since the particles have effectively identical sizes and nominally identical particle counts, a proportionality correction was applied to correct for the intrinsic variation in the measurement due to refractive index differences using equation S5.

$$\frac{No\ Dye\ PNC}{Dyed\ PNC} = \frac{FITC\ Unknown\ PNC\ Corrected}{FITC\ Unknown\ PNC\ Measured} \quad \textbf{(S6)}$$

The FITC-like particle count was made using the AF4-MALS methodology (Measured). Then the ratio of the No Dye:Dyed measurements were used as a proportionality correction factor to provide the corrected particle number count for the FITC-like VCN.

In an alternative approach, the No Dye sample was considered the true (i.e. reference) particle number count. The refractive index used in the calculation was that of pure polystyrene latex 1.5915. Then the measurement of the Dyed sample was adjusted to match the particle number count of the No Dye sample and the refractive index was modified iteratively until the particle number counts matched. The result of this manipulation gave a Dyed refractive index of 1.6115. This overall implies that the addition of dye to the polystyrene results in a significant change to the refractive index.

## 3. Results

**Table S5:** Comparison of Percent Difference from Consensus Values to Expanded Uncertainty for Measured Number Concentrations and Diameters of VCN Suspensions

| Technique | VCN-100 | | VCN-200 | | VCN-500 | |
|---|---|---|---|---|---|---|
| | **% Diff for Size** | **% Diff for Concentration** | **% Diff for Size** | **% Diff for Concentration** | **% Diff for Size** | **% Diff for Concentration** |
| Size: TEM / Number Conc.: Consensus Value | **0%**±13% | **0%**±8% | **0%**±13% | **0%**±6% | **0%**±12% | **0%**±5% |
| PTA | **-1%**±4% | **-32%**±22% | **-7%**±2% | **-8%**±22% | **-12%** ±4% | **-39%** ±25% |
| MRPS | **+6%** ±14% | **-42%**±15% | **-9%**±12% | **-21%**±15% | **-15%** ±5% | **-12%** ±15% |
| AF4-MALS | **-19%** ±9% | **+81%**±3% | | | | |
| VC | | | N/A | **+16%**±23% | N/A | **-10%** ±13% |

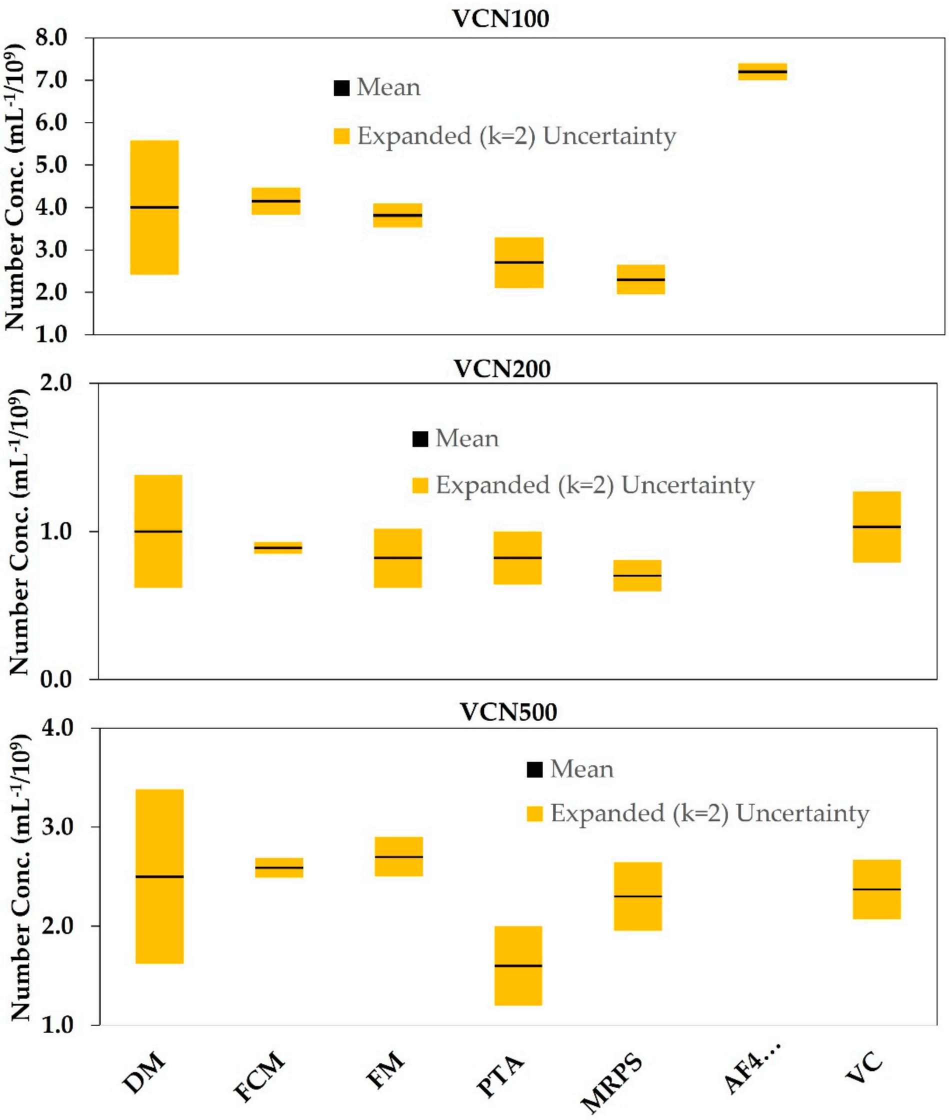


**Figure S3:** Mean Number Concentration of VCNs in Stock Suspensions, Measured Using Each Technique. The error bars show the estimated expanded (*k*=2) uncertainties for the technique values.

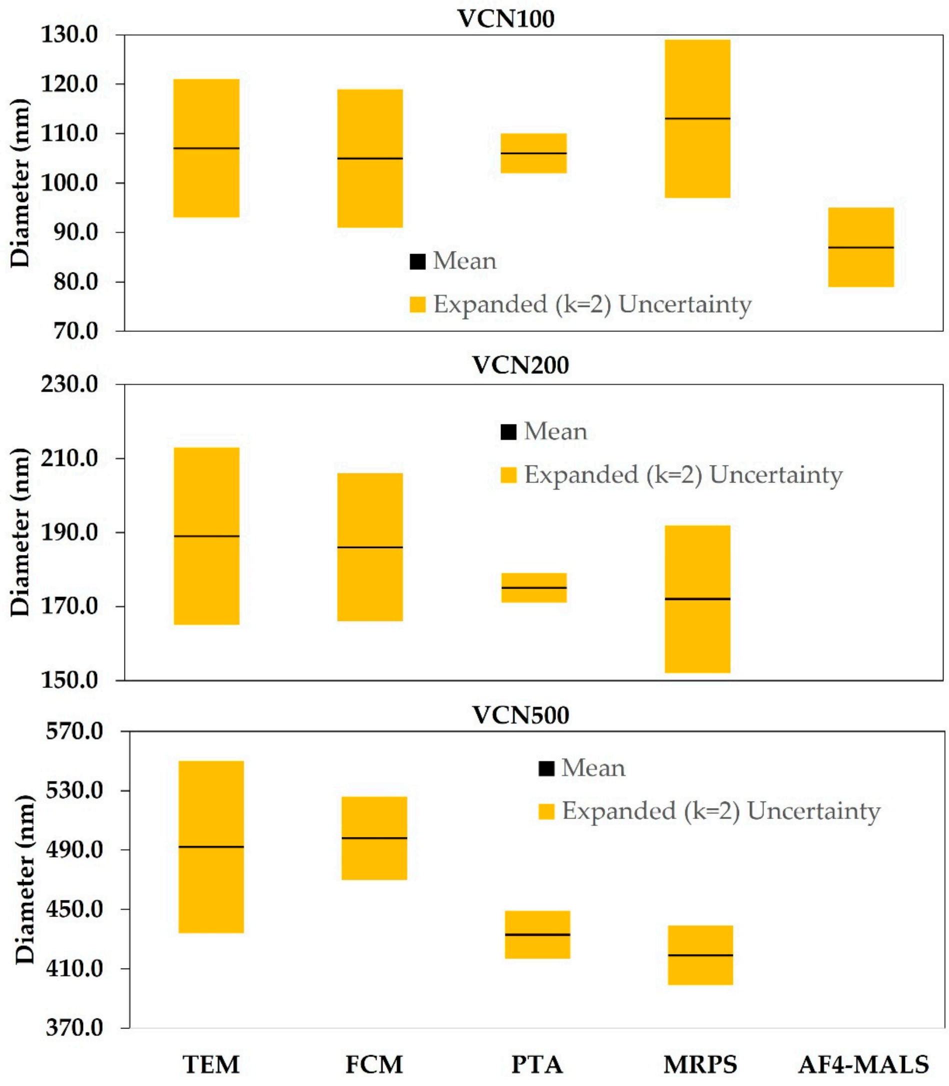


**Figure S4:** Mean Measured Diameters for the VCNs Using Each Technique and TEM. The error bars show the estimated expanded ($k$=2) uncertainties for the TEM and technique values.

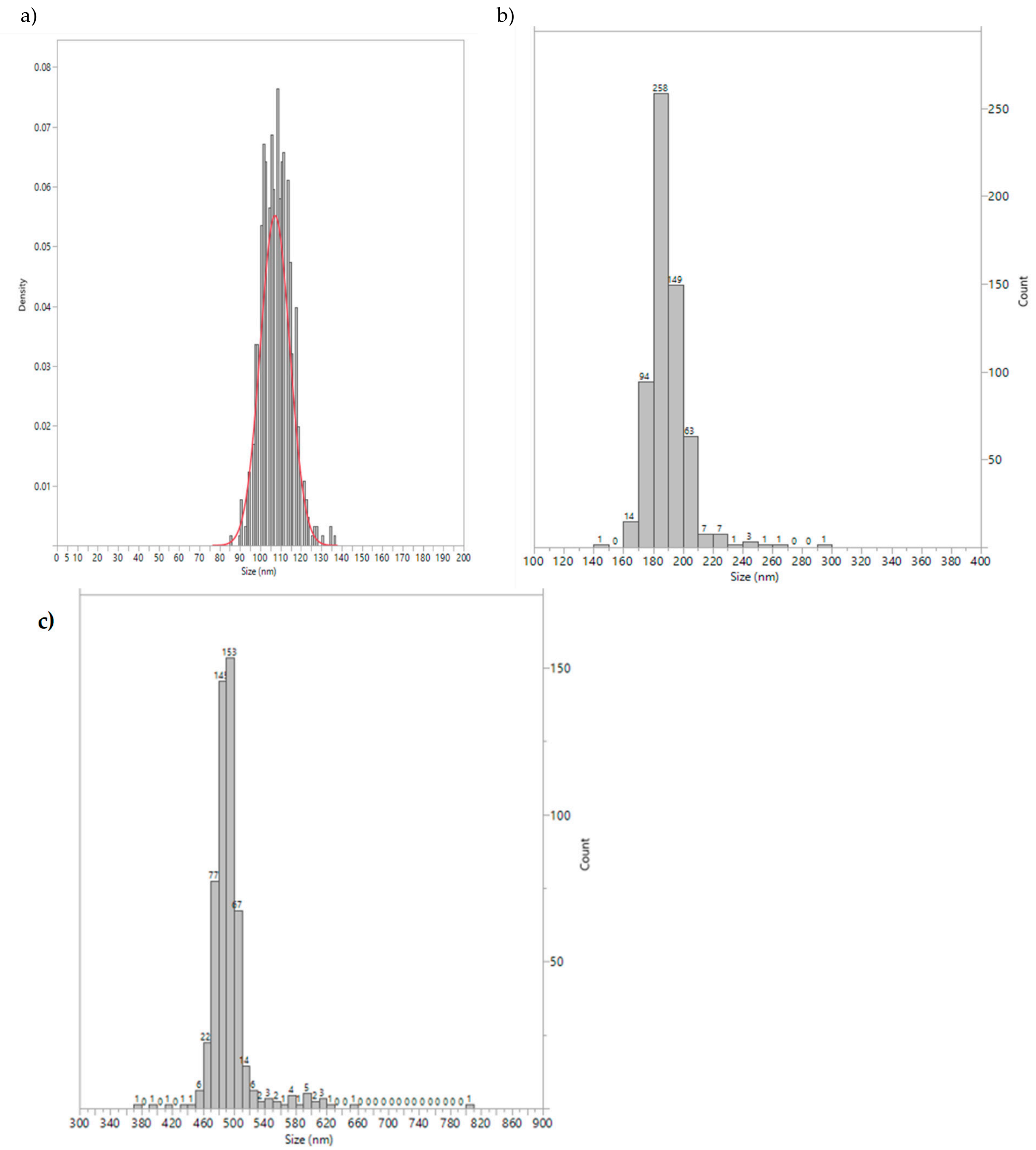


**Figure S5:** TEM Size Distributions of a) VCN-100, b) VCN-200 and c) VCN-500 spheres.

**NIST Disclaimer:** Certain commercial equipment, instruments and materials are identified in this paper to specify an experimental procedure as completely as possible. In no case does the identification of particular equipment or materials manufacturer imply a recommendation or endorsement by the National Institute of Standards and Technology nor does it imply that the materials, instruments, or equipment are necessarily the best available for the purpose. This manuscript is a contribution of NIST, and therefore is not subject to copyright in the United States.

1. Caicedo, J., Cooper, S., Heigwer, F. et al. Data-analysis Strategies for Image-based Cell Profiling. **2017**, *Nat. Methods*, 14, 849–863. https://doi.org/10.1038/nmeth.4397.
